# Body mass index and mortality in chronic obstructive pulmonary disease: A dose–response meta-analysis

**DOI:** 10.1097/MD.0000000000004225

**Published:** 2016-07-18

**Authors:** Yibin Guo, Tianyi Zhang, Zhiyong Wang, Feifei Yu, Qin Xu, Wei Guo, Cheng Wu, Jia He

**Affiliations:** a Department of Health Statistics; b Department of Information, Changhai Hospital, Second Military Medical University, Shanghai, China.

**Keywords:** body mass index, chronic obstructive pulmonary disease, dose–response meta-analysis, mortality

## Abstract

The aim of this study is to summarize the evidence on the dose–response relationship between body mass index (BMI) and mortality in patients with chronic obstructive pulmonary disease (COPD).

We performed a systemic literature search in PubMed, Embase, and Web of Science for relevant studies that were published until June 2015. A random effects meta-analysis was used to estimate the pooled relative risks (RRs) of all-cause mortality in COPD patients with normal weight compared with those who were underweight, overweight, or obese. In addition, a dose–response meta-analysis was conducted to explore the dose–response relationship between BMI and all-cause mortality in COPD patients.

A total of 17 observational studies involving 30,182 COPD patients among 285,960 participants were included. Compared with the reference category, the RRs of underweight, overweight, and obese individuals were 1.40 (95% confidence interval (CI), 1.20–1.63), 0.80 (95% CI, 0.67–0.96), and 0.77 (95% CI, 0.62–0.95), respectively. A significant nonlinear relationship between BMI and mortality of COPD patients was found by using a random effects model. COPD patients with BMI of <21.75 kg/m^2^ had a higher risk of death. Moreover, an increase in the BMI resulted in a decrease in the risk of death. The risk of death was lowest when BMI was 30 kg/m^2^ (RR = 0.69; 95% CI, 0.53–0.89). The BMI was not associated with all-cause mortality when BMI was >32 kg/m^2^.

Our findings indicate that overweight is associated with a lower risk of all-cause mortality among patients with COPD whereas underweight is associated with a higher risk of all-cause mortality in these patients. However, there is limited evidence to support the association between obesity and the risk of all-cause mortality in patients with COPD.

## 1 Introduction

Chronic obstructive pulmonary disease (COPD) is a major public health problem, and its prevalence and mortality are increasing throughout the world. In addition, the prevalence and mortality of COPD are expected to increase over the coming decades.^[1]^ It is known that obesity is associated with higher mortality in the general population. A recent meta-analysis^[2]^ showed that obesity was significantly associated with higher all-cause mortality compared with normal weight in the general population. However, a meta-analysis performed by Cao et al^[3]^ indicated that overweight and obese patients with COPD had a lower risk of all-cause mortality. This phenomenon is known as the “obesity paradox,” which is defined as an inverse relationship between survival and obesity and has been observed in various chronic diseases, including type II diabetes mellitus,^[4]^ stroke,^[5]^ and chronic kidney disease.^[6]^ To date, the relationship between obesity and mortality in patients with COPD has been controversial^[7]^ but evidence has indicated a dose–response relationship between BMI and mortality. Several recent studies on this topic have reported that the quantitative dose–response relationship is still unknown. Therefore, we searched for recently published studies and performed a dose–response analysis to explore the dose–response relationship between BMI and mortality in patients with COPD.

## 2 Materials and methods

### 2.1 Search strategy and selection criteria

Observational studies (retrospective reviews, retrospective cohort studies, and prospective cohort studies) on BMI and mortality in patients with COPD were included in this meta-analysis. No language restriction was imposed.

Two investigators (YG and TZ) conducted a systematic literature search by using the electronic databases PubMed (from 1965 to June 2015), Embase (from 1965 to June 2015), and Web of Science (from 1986 to June 2015). Searches were performed using Medical Subject Heading terms and free keywords (“COPD” OR “Chronic Obstructive Pulmonary Disease” AND “Body Mass Index” OR “Overweight” OR “Obese” OR “Obesity”). Moreover, a manual search of the reference lists of the obtained articles was performed.

Eligible studies met the following inclusion criteria: (a) they were observational studies (retrospective reviews, retrospective cohort studies, and prospective cohort studies); (b) the study population included individuals diagnosed with COPD; (c) the outcome was all-cause mortality; (d) the studies reported either the relative risk (RR), odds ratio (OR), or hazard ratio (HR), together with the 95% confidence interval (CI) for the relationship between BMI and mortality in COPD, or the total number of patients with events. The studies included in the dose–response analysis should have three or more BMI categories, and each BMI category should have an effect value. The studies with less than two BMI categories were not included in the dose–response analysis. The studies were selected independently by two investigators (YG and TZ).

### 2.2 Data extraction and quality assessment

The following information was extracted from each eligible study (when available): the names of the first author; year of publication; countries where the study was conducted; proportion of male participants; number of years of follow-up; sample size; BMI and either RR, OR, or HR, together with the 95% CI for each BMI category; number of cases; and sample size for each BMI category. The adjusted RRs were extracted in case the studies provided both crude and adjusted RRs. If a study reported more than one multivariable-adjusted effect estimates, we chose the result fully adjusted for potential confounding variables.

Quality assessment was conducted using the 9-star Newcastle–Ottawa scale (NOS).^[8]^ In our study, we defined high-quality studies as the studies with an NOS score of ≥6. CW examined and adjudicated all the extracted information and results of quality assessment independently after data extraction and assessment. Institutional review board approval and patient consent were not required for this meta-analysis of observational studies.

### 2.3 Statistical analysis

In this meta-analysis, the number of patients with events, total participants, and adjusted or crude RRs, HRs, and the 95% CI reported by the selected studies were extracted. First, we performed three meta-analyses to estimate the pooled effects between normal weight and underweight, overweight, and obesity, respectively. The random effects model was used to estimate the pooled RR and its 95% CI. Because a normal BMI of 18.5 to 25.0 kg/m^2^ is recommended by the International Agency for Research on Cancer to maintain a healthy condition,^[9]^ we selected the average normal BMI as the reference (BMI = 21.75). In case a study defined normal BMI as 20 to 25 rather than 18.5 to 25, we chose the former category as the reference.

For the dose–response meta-analysis, the mean or median in each BMI category, when available, was set as the mid-point in each category. When the highest or lowest category was open ended, we assumed that the open-ended interval length was the same as the adjacent interval. A nonlinear relationship between BMI and mortality in COPD was used when performing the dose–response analysis. To derive the dose–response curve, BMI was modeled using restricted cubic splines with three knots in fixed percentiles (10%, 50%, and 90%) of the distribution. We estimated the *P*-value of the null hypothesis that the coefficient of the second spline was equal to zero. The details of the methods used have been described by Larsson and Orsibi.^[10,11]^

R software version 3.1.2 with doseresmeta and metafor packages was used to obtain the pooled analysis, dose–response analysis, and to retrieve the plots. All statistical tests were two-sided. A *P*-value <0.05 was considered statistically significant. The *I*^2^ test and *Q* test were used to explore the heterogeneity among the studies.^[12]^ The Egger's regression test^[13]^ was performed to assess publication bias. In case of significant publication bias, a nonparametric trim and fill method^[14]^ was used to explore the adjusted pooled effects.

A sensitivity analysis was performed by excluding the studies one at a time and observing any substantial changes in the pooled results.

## 3 Results

### 3.1 Description of the selected studies

Of the 9405 articles identified via literature search, 13 observational studies^[15–27]^ involving 285,960 participants and 30,182 cases were included in this meta-analysis on the basis of the selection criteria. Of these, 11 studies^[15–25]^ contained at least 3 BMI categories and therefore were included in the dose–response analysis. The duration of follow-up ranged between 1 and 17 years.

**Figure 1:**
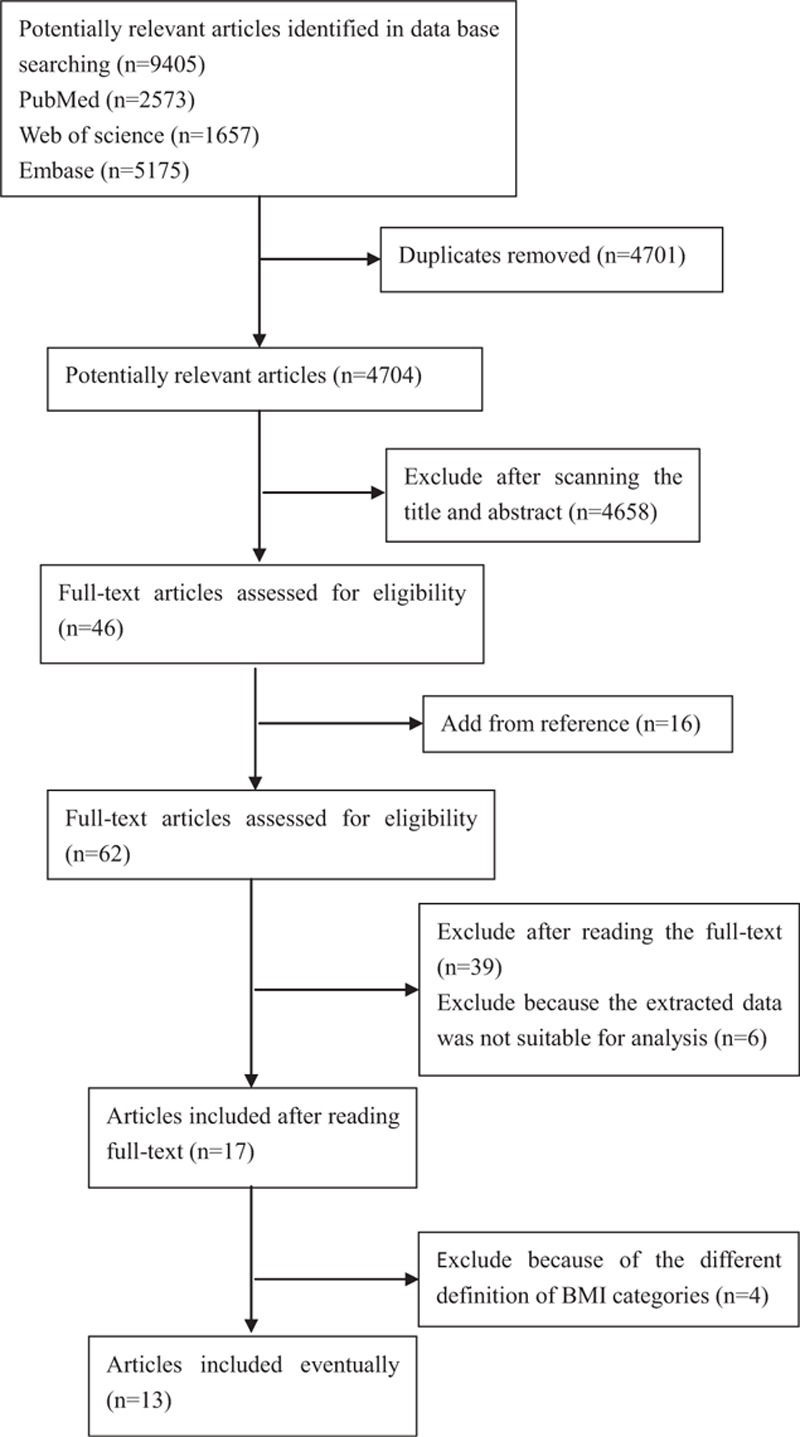
Literature search and study selection.

The search process is depicted in Fig. 1. The general characteristics of the selected studies are presented in Table 1.

**Table 1:**
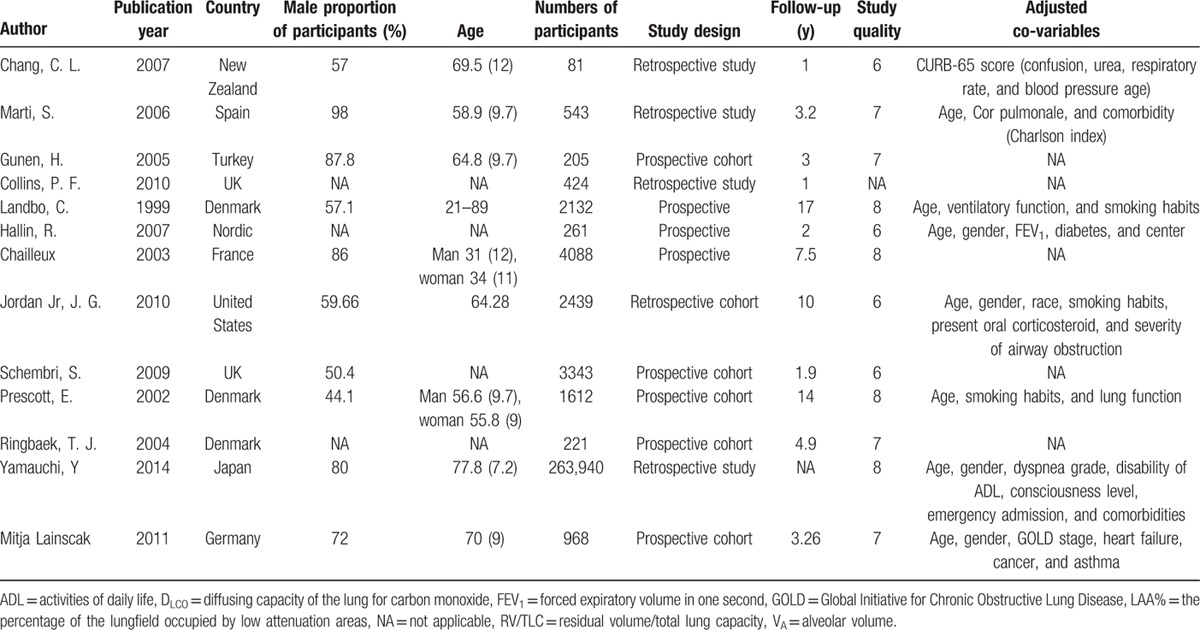
Characteristics of studies included in meta-analysis.

### 3.2 Effects of BMI on mortality in COPD

**Figure 2:**
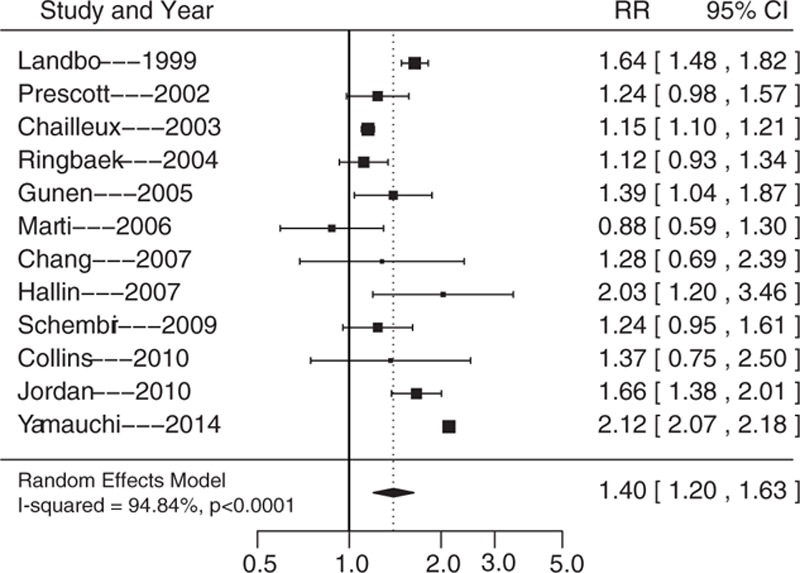
Summary of relative risks of all-cause mortality in underweight COPD patients. COPD = chronic obstructive pulmonary disease.

**Figure 3:**
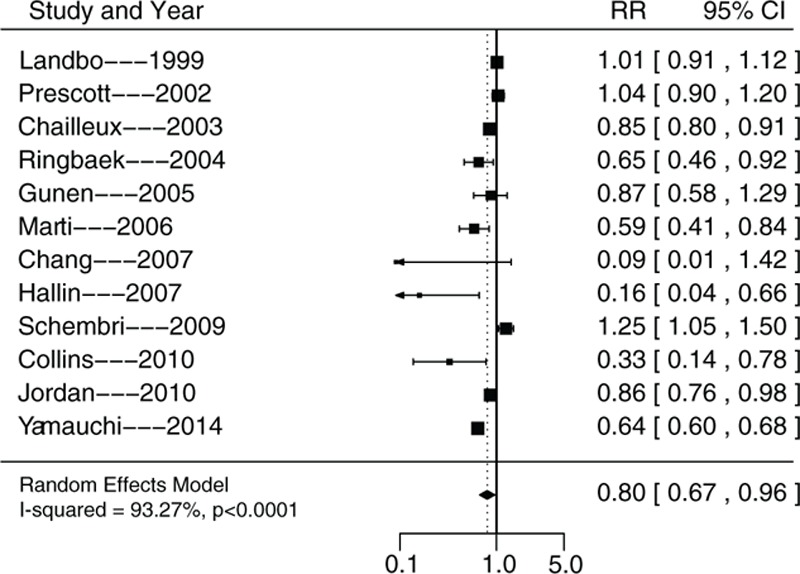
Summary of relative risks of all-cause mortality in overweight COPD patients. COPD = chronic obstructive pulmonary disease.

**Figure 4:**
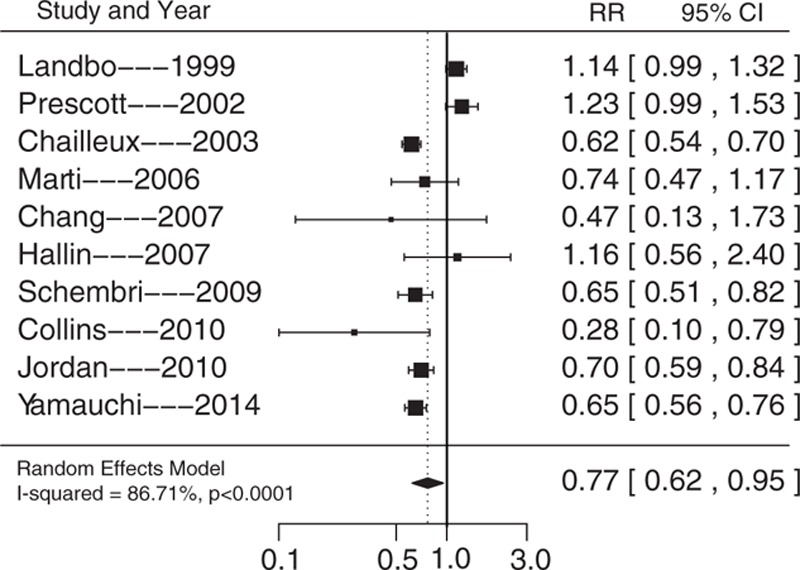
Summary of relative risks of all-cause mortality in obese COPD patients. COPD = chronic obstructive pulmonary disease.

Compared with normal weight, the RRs of underweight, overweight, and obese individuals were 1.40 (95% CI, 1.20–1.63; *P* <0.0001), 0.80 (95% CI, 0.67–0.96; *P* <0.0001), and 0.77 (95% CI, 0.62–0.95; *P* = 0.0162), respectively (Figs. 2–4). The heterogeneity test found I^2^ values of 94.84%, 93.27%, and 86.71%, for underweight, overweight, and obesity, respectively, and all the *P*-values obtained in the *Q* statistic test were <0.0001.

### 3.3 Dose–response analysis

**Figure 5:**
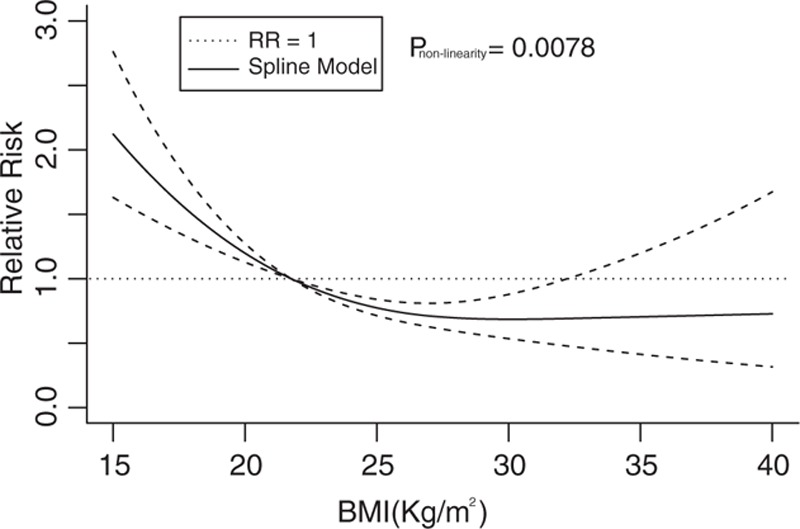
Dose–response relationship between BMI score and risk of all-cause mortality. BMI = body mass index.

We found a significant nonlinear relationship (*P* = 0.0078) between BMI categories and mortality in patients with COPD using random effects models. Figure 5 shows that the COPD patients whose BMI was less than the median of the normal weight category (<21.75 kg/m^2^) had a higher risk of death. In addition, an increase in the BMI resulted in a decrease in the risk of death. The risk of death was lowest (RR = 0.69; 95% CI, 0.53–0.89) when BMI was 30 kg/m^2^. The increase in BMI was no longer a protective factor for the risk of death when BMI reached >32 kg/m^2^, which was defined as obesity. The dose–response curve was “U” shaped.

### 3.4 Subgroup analysis

**Figure 6:**
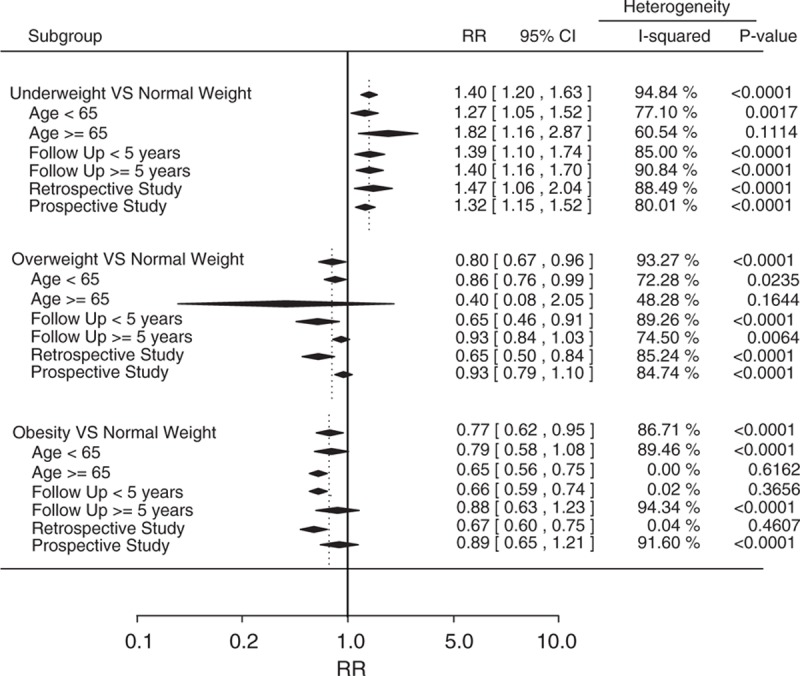
Subgroup analysis stratifying by age, follow up, and study design.

To explore the sources of heterogeneity, we performed a subgroup analysis by stratifying the BMI categories by age (<65 years and >65 years), follow-up period (>5 years and <5 years), and study design (perspective and retrospective study). As shown in Fig. 6, compared with normal weight, the effect of underweight was similar in each subgroup. For COPD patients who were overweight or obese, the association between higher body weight and lower mortality was not significant when the follow-up period was >5 years or the design of the studies was prospective. The follow-up period of the prospective studies included in our meta-analysis was >5 years. Therefore, this association may occur only with a relatively short (5-year) follow-up and for a high BMI. We also found that obesity was associated with a lower risk of all-cause mortality in COPD patients >65 years. However, no relationship between obesity and mortality was found for COPD patients <65 years. In addition, the subgroup analysis to some extent decreased the heterogeneity between the studies.

### 3.5 Publication bias

Egger's test indicated the absence of publication bias for underweight, overweight, and obesity compared with normal weight. The *P*-values in this test were 0.38, 0.20, and 0.73, respectively.

### 3.6 Sensitivity analysis

To determine sensitivity, we excluded one study at a time. In the comparison between normal weight, underweight, and overweight, the RRs and 95% CIs did not change substantially after removing one study at a time. However, after removing several single studies out one by one, the relationship between normal weight and obesity become not significant. This result was in agreement with the dose–response analysis results.

## 4 Discussion

In this study, we updated the evidence and conducted a dose–response meta-analysis to elucidate the relationship between BMI and mortality in COPD patients. Our analysis indicated that underweight significantly increased the risk of all-cause mortality by 40%. By contrast, overweight and obesity were significantly associated with a decrease by 20% and 23% in the risk of all-cause mortality in patients with COPD, respectively. However, the relationship between obesity and higher risk of mortality was not stable so we removed several studies^[15,25]^ one by one. Furthermore, the dose–response analysis indicated that the relationship between BMI and mortality in COPD patients was nonlinear. Compared with a BMI of 21.75 kg/m^2^, BMI <21.75 kg/m^2^ increased the risk of death in these patients whereas BMI >21.75 kg/m^2^ significantly decreased this risk. As the BMI was increasing, the RR was larger and larger than BMI with 21.75 kg/m^2^. When BMI reached 30 kg/m^2^, the RR was largest. The RR decreased for values >30 kg/m^2^, and when the BMI was >32 kg/m^2^, there was no significant difference in the risk of mortality compared with patients with a BMI of 21.75 kg/m^2^.

BMI is relatively easy to measure and has become a part of the BODE (BMI, Obstruction, Dyspnea, and Exercise capacity) index which is the most widely used tool to predict mortality in COPD patients.^[28]^ The BODE index is calculated as the sum of the BMI, obstruction, dyspnea, and exercise capacity scores and ranges between 0 and 10. A higher BODE index indicates a greater risk of death. The BMI in BODE is scored as 0 and 1 and its cutoff value is 21 kg/m^2^. However, the result of the dose–response analysis indicated that the relationship between BMI and mortality was non-linear and the effect of BMI on mortality was different in each category. Therefore, a more consistent scoring system for BMI should involve a division into three categories: a score of 2 for a BMI <21 kg/m^2^, 0 for a BMI of 21 to 30 kg/m^2^, and 1 for a BMI >30 kg/m^2^.

Our results suggest that underweight is associated with a higher risk of all-cause mortality in COPD patients. The reasons for this are still unclear. However, several hypotheses have been put forward to interpret this phenomenon, including respiratory muscle weakness, impaired gas exchange, impaired immune response,^[27]^ and loss of metabolically and functionally active fat-free mass (FFM).^[29]^ Underweight patients have an increased frequency of exacerbation, which leads to a faster decline in FEV_1_, impaired quality of life, and high mortality.^[19]^ On the contrary, obese patients with COPD may receive medical attention earlier than normal weight patients possibly because obesity is also associated with dyspnea.^[30]^

With regard to obesity, our sensitivity analysis indicated that the association between body weight and all-cause mortality was not significant. Furthermore, Jordan and Mann^[20]^ observed an increase in mortality because of respiratory disease in extremely obese COPD patients. Therefore, there is limited evidence to demonstrate a close relationship between obesity and mortality. For this reason, further studies are needed to elucidate this relationship.

This study has several limitations. First, although several included studies were adjusted for potential covariates, we could not rule out the influence of other confounding factors. Second, different studies used different BMI category in particular for the definition of underweight. These differences may underestimate the risk of death in underweight patients with COPD. For this reason, we conducted a dose–response analysis to estimate the relationship between BMI and mortality accurately and to eliminate the inconsistency in the definition of the BMI categories. Third, this meta-analysis indicated a significant heterogeneity between the studies. This heterogeneity might be attributed to differences in sample size, duration of follow-up, disease severity, cutoff value for underweight, sex ratio, and the mean age of the population. Therefore, we conducted a subgroup analysis to explore the source of heterogeneity.

## 5 Conclusion

In summary, underweight increased the risk of mortality in patients with COPD whereas overweight decreased this risk. Furthermore, we found a nonlinear dose–response relationship between BMI and mortality in COPD patients. The risk of mortality was lowest for a BMI of 30 kg/m^2^. In this context, nutritional support may improve the prognosis of COPD patients in clinical practice.
